# Determination of the Elastic Properties of Tomato Fruit Cells with an Atomic Force Microscope

**DOI:** 10.3390/s130912175

**Published:** 2013-09-11

**Authors:** Artur Zdunek, Andrzej Kurenda

**Affiliations:** Institute of Agrophysics, Polish Academy of Sciences, Doswiadczalna 4, Lublin 20-290, Poland; E-Mail: a.kurenda@ipan.lublin.pl (A.K.)

**Keywords:** AFM, indentation, tomato, cell, Young's modulus, Hertz, Sneddon, stiffness, texture, biomechanics

## Abstract

Since the mechanical properties of single cells together with the intercellular adhesive properties determine the macro-mechanical properties of plants, a method for evaluation of the cell elastic properties is needed to help explanation of the behavior of fruits and vegetables in handling and food processing. For this purpose, indentation of tomato mesocarp cells with an atomic force microscope was used. The Young's modulus of a cell using the Hertz and Sneddon models, and stiffness were calculated from force-indentation curves. Use of two probes of distinct radius of curvature (20 nm and 10,000nm) showed that the measured elastic properties were significantly affected by tip geometry. The Young's modulus was about 100 kPa ± 35 kPa and 20 kPa ± 14 kPa for the sharper tip and a bead tip, respectively. Moreover, large variability regarding elastic properties (>100%) among cells sampled from the same region in the fruit was observed. We showed that AFM provides the possibility of combining nano-mechanical properties with topography imaging, which could be very useful for the study of structure-related properties of fruits and vegetables at the cellular and sub-cellular scale.

## 1. Introduction

Mechanical properties and texture have important roles in determining the quality of fruits and vegetables [1]. Structure-related properties of plants could be considered at various length scales: at macroscale—tissues' spatial variability [2], at microscale—cell size and orientation [3–5], at the submicro and nanoscale—cell walls [6–8] and middle lamella [9] composition and at nanoscale—individual polysaccharide structures and linkages in cell walls [10,11] Up to now, plenty of studies which have used a continuum approach have been performed on the mechanical properties at the macro-scale, while studies at the microscopic and nanoscopic levels have been constrained by the lack of suitable technology to conduct mechanical manipulation at such a length scale [12]. Investigation of the micro and nano-mechanics of plants is important because the composition at this level is very dynamic, e.g., during postharvest maturation of fruits [13]. On the other hand, cells are the basic units responsible for the mechanical behaviour of the whole plant and therefore most often they are considered as the starting point for the structure-related biomechanical models [14].

Up to now a few techniques have been developed to investigate the mechanics of single cells, such as compression between two parallel plates [15] or micro- and nano-indentation methods [16]. The basic micromanipulation technique for the compression of single cells was described by Mashmoushy *et al.* [15]. The cell is compressed between the flat end of an optic fibre and a glass surface. Compression is performed in a relatively large force range (10–1000 μN) and with strain of up to about 0.3 [12], often ended with the cell bursting [17]. Micro-indentation can be used to measure the mechanical properties of single cells or tissue. Indentation is performed by a flat or rounded indenter, a few micrometres in diameter (1–5 μm). In turn, the indentation depth is comparable to or larger than the cell wall thickness and the force is in a range of 1–100 μN [18]. A device that allows automation of micro-indentation measurements is the cellular force microscope (CFM) [19]. CFM, which is an assembly of commercially available components, is a micrometre resolution system capable of applying forces from the submicronewton to the milinewton range using a hemispherical probe of 1–3 μm in diameter.

Recently, an atomic force microscope (AFM) has been used for nano-indentation of biological materials, including living cells. AFM is capable of applying forces in the range from subnanonewtons to ∼100 nN with an indentation depth in the range of several nanometres to a few micrometres. AFM combines imaging of topography with nanometre resolution and sensing of the nano-mechanical properties of the sample. Nano-indentation with an AFM could be utilized in three general ways. Single force curve analysis involves approaching the AFM cantilever at a chosen single point and the measurement of the cantilever deflection during indentation in the material [20]. Force volume, introduced by Radmacher *et al.* [21], involves collecting a matrix of the force curves across the sample surface that are individually analysed, allowing spatial reconstruction of topographic maps of mechanical properties. Since the tip moves from point to point across the surface, the AFM tip being completely detached from the surface before moving to the next point, the problem of lateral forces associated with contact imaging of soft surfaces is avoided. The third and latest approach is a combination of topographic images with permanent monitoring force curves at each point of the image, which allows the user to obtain a fine map of mechanical properties, like a modulus of elasticity and adhesion [22,23]. The sample elasticity is determined by fitting the force-indentation curve to a mathematical model of the contact between tip and sample. The Hertz model is the most commonly used one, with the assumption that the sample is a linearly elastic isotropic solid occupying an infinitely extending half space [24]. The Hertz model enables the determination of the sample Young's modulus (elastic modulus *E*) when the indenter geometry and Poisson ratio of the sample are known. The nano-indentation of individual cells with an AFM has been recently applied to living plant cells to measure mechanical properties of suspended grapevine cells cultured in liquid medium [25], the primary cell wall of shoot apical meristems [26,27], rosette leaves [28] and epidermal cells of living roots [29] of *Arabidopsis thaliana*. Most of the studies applied low indentation (<100 nm) to cells, which produces force in the range of nanonewtons, however in the work of Fernandez *et al.* [29] indentation of 1.5 μm was reached. The idea of low indentation is to avoid turgor and the support effect and to probe the stiffness of the cell wall only. A wide range of the AFM cantilevers have been used in the above studies, with a nominal spring constant in the range of 0.06–2 N/m and tip radii of curvature in the 20–5,000 nm range. A typical assumption was that the Poisson ratio of cells is 0.3 or 0.5.

The above applications of AFM to examine plant cell nano-indentation were aimed at studying plant morphogenesis and this technique has not been used for living cells extracted directly from naturally growing fruits. Therefore the aim of this work is to measure the elastic properties of cells isolated from tomato mesocarp with an atomic force microscope in order to assess the usefulness of this method for further study of fruit texture at the micro and nano-scales. Since cell wall nano-structure is assembled from polysaccharides of several nanometers of diameter and that a typical AFM tip radius curvature is of the same size, the indention process should be significantly different from indentation performed by tips with larger curvature (micrometers). The influence of the tip radius on indentation process and apparent elasticity of plant cells has not been discussed yet, therefore the second aim was to evaluate an effect of the AFM tip geometry on the apparent elasticity of tomato cells.

## 2. Experimental Section

### 2.1. Mechanical Isolation of Tomato Mesocarp Cells

**Figure 1.**
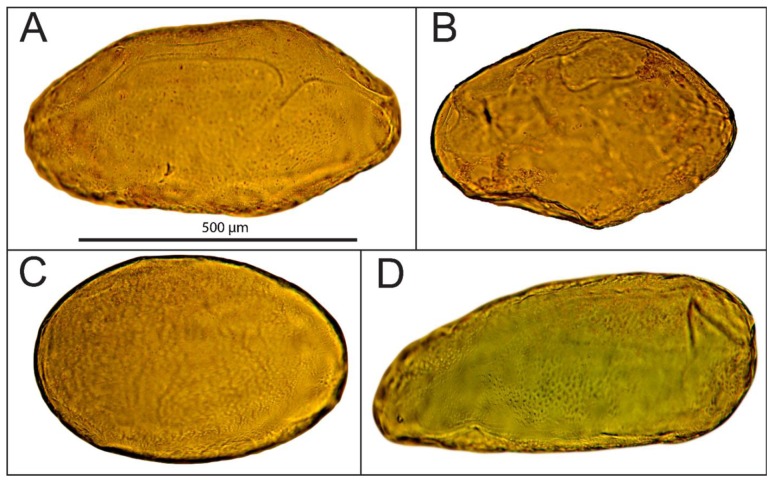
Viable (**A)** and (**C**), and nonviable (**B**) and (**D**) cells extracted from tomato mesocarp. (**A**) a cell evaluated visually as viable according to the integrity of the cell wall and protoplast, (**B**) a cell with a disintegrated cell wall, (**C**) viable cell from Evans Blue test, (**D**) nonviable cell from Evans Blue test.

**Figure 2.**
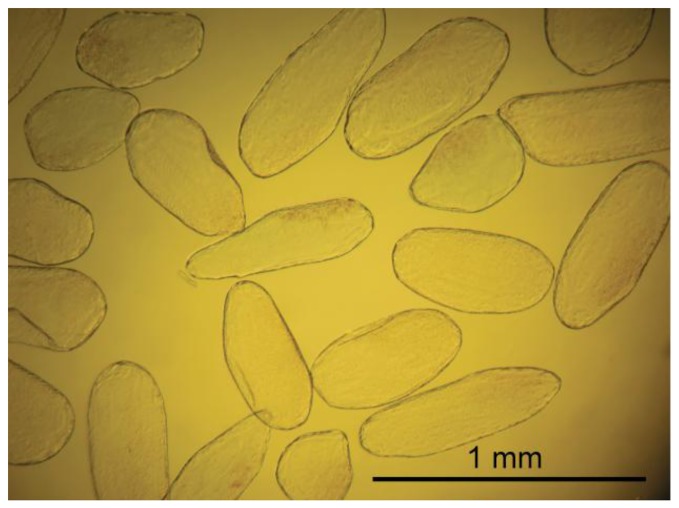
Suspension of viable cells from tomato mesocarp ready for AFM nano-indentation.

Cells were extracted from the mesocarp of fully ripe tomato fruits (*Lycopersicon esculentum* cv. Admiro). The procedure of cell isolation consisted of mild maceration of a fragment of the mesocarp and filtration of the homogenate in an Murashige-Skoog (MS) solution (Sigma-Aldrich, St. Louis, MO, US). A fragment of mesocarp (1 × 3 cm) without epidermis, egzocarp and endocarp was cut into smaller pieces with a thickness of about 3 mm. Pieces of tissue were homogenized gently for about five minutes with a pestle in the MS medium. The homogenate was filtered twice with 500 mL MS medium each; first through 1 mm mesh nylon filters to separate larger fragments of tissue and next through 100 μm nylon mesh to remove remnants of damaged cells. Filtered material consisted mainly of single cells. Cell viability was determined in two stages. In the first stage, the cells were evaluated visually under a microscope for the integrity of the cell wall and protoplast (Figure 1A,B). Those classified as viable cells were separated from the solution and subjected to the second stage of testing using the method presented by Castro-Concha *et al.* [30] using Evans Blue (Figure 1C,D). Cells that in the second stage of the evaluation were classified as viable were gently washed with a solution of MS and deposited on poly-L-lysine coated microscope slides (Menzel-Gläser; Figure 2).

### 2.2. AFM Indentation

Cells immobilized on the glass substrate in the MS medium were indented with AFM (Bioscope Catalyst II, Bruker, Billerica, MA, USA). Two AFM probes were used:

1. A ScanAsyst-Fluid probe with a nominal spring constant of 0.7 N/m and a nominal tip radius *R* = 20 nm (Bruker, www.brukerafmprobes.com), hereafter called the sharp probe. The probe has a pyramidal non-symmetric shape with an average half cone opening angle α = 19°.
2. Pyrex-Nitride probe (type CP-PNP-BSG) with a nominal spring constant of 0.32 N/m and with single spherical colloid of radius *R* = 10,000 nm attached to the cantilever (sQube, Bickenbach, Germany, www.sqube.de), hereafter called the bead probe.

The tip calibration procedure involved two steps. Both of them were performed in the MS solution. First, the deflection sensitivity of the cantilever was determined on the glass substrate assuming that the surface has infinite hardness in relation to plant cells. The deflection sensitivity, expressed in V/nm, allowed for the conversion of a voltaic signal from the AFM photodetector into a cantilever deflection in metric units. This calibration was performed in ten repetitions. Then, an averaged value was used. The second calibration was the determination of a spring constant *k* using the thermal tune method (simple harmonic oscillator available in the Nanoscope 8.10 software provided with Bioscope Catalyst II). This calibration was performed in the withdraw position of the AFM probe, in five repetitions. Then, an averaged value was used. Both calibration steps were repeated when, due to whatever reason, a laser position on the cantilever had been changed.

**Figure 3.**
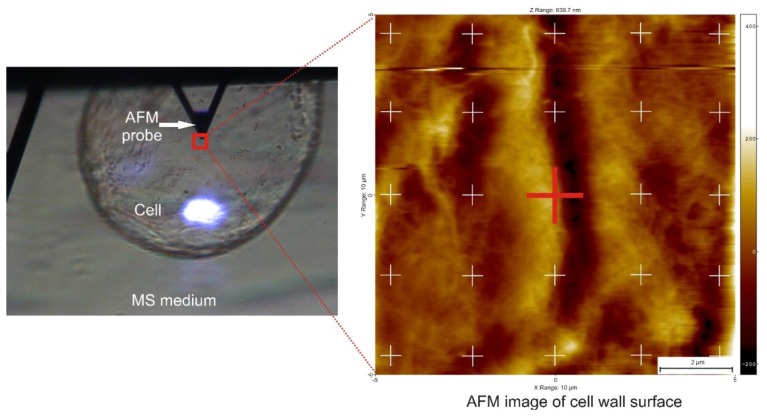
A typical experimental view during nano-indentation of a naturally grown tomato cell with AFM. An example of a topography image of a tomato cell wall, which is possible to obtain with a sharp probe of radius *R* = 20 nm, is shown to the right. The white crosses present a matrix of points of the mechanical measurements for tip of radius *R* = 20 nm. The red cross presents an exemplary place of indentation with the tip of radius *R* = 10,000 nm.

Before the AFM probe was engaged with the sample, a cell firmly fixed to the substrate was searched for using an optical preview in the AFM. It was visible that some cells swam in the buffer when the probe was moved in the horizontal direction, however some cells were attached firmly to the substrate. The later ones were used for mechanical testing. When such a cell was found, the AFM probe was positioned just above the cell (Figure 3). The ScanAsyst mode which directly controls the interaction force between sample and the probe to obtain AFM image and so called point and shoot method to measure local mechanical properties were used. Before engaging the probe with the cell surface, a scan size was set to 0 nm and a set point to 0.3 V. Then the probe was engaged automatically with the cell. Further steps were slightly different for the two probes used. For the AFM probe with a radius of curvature *R* = 20 nm, when the probe was engaged with the sample, the set point was set back to −10 V, which caused retraction of the probe from the cell surface. Then, in the retract position, a scanning size of 10 μm was set and a blank image was scanned. This preserved the cell from possible alteration by lateral movement of the AFM probe. Then, the set point was set to 1 V, which caused extension of the AFM probe to the sample and 25 points in a regular matrix were marked (5 × 5 points) on the scan (Figure 3). At each point, ten indentations were performed. For the AFM probe with a radius of curvature *R* = 10,000 nm, directly after the probe has been engaged, 250 force curves were collected from the same point. For both probes, a relative force trigger >100 nN, an approach size of 10 μm and forward-backward velocity of 20 μm/s were set. Deflection error (V) *vs.* Z-height (nm) was recorded. Force-separation curves and Young's modulus estimation were performed in the SPIP™ (Image Metrology A/S, Hørsholm, Denmark) software as:

(1) Force=k×x×deflection sensitivity

where *k* is the spring constant (N/m) from the thermal tune method and *x* is the cantilever deflection (V). Deflection sensitivity (nm/V) was obtained from calibration on the hard surface:

(2) Separation=ΔZ−height+Δcantilever deflection

Force-separation curves where also exported to the text format and analysed in order to evaluate stiffness of cells. Stiffness was calculated in Matlab (MathWorks, Natick, MA, USA).

### 2.3. Determination of Elastic Properties

**Figure 4.**
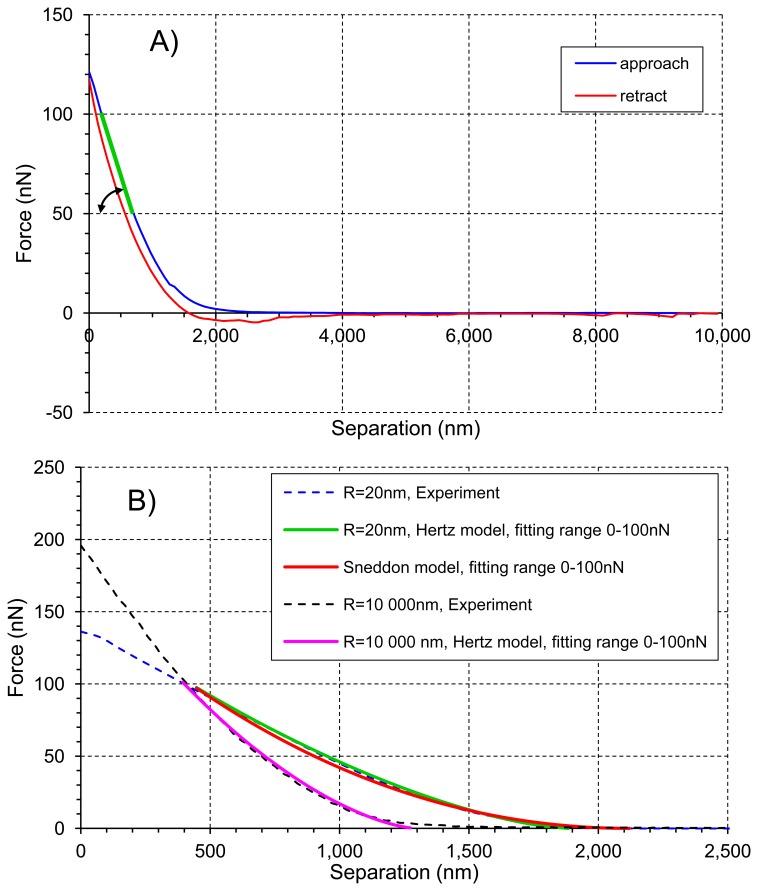
(**A**) Representative force-separation curve (approach and retract) obtained in the experiment on tomato suspension cell in liquid with a sharp probe (*R* = 20 nm). Stiffness was measured as the slope of the line drawn between 50 nN and 100 nN (green line). (**B**) Representative approach curves obtained with two probes: a sharp probe (*R* = 20 nm), blue dashed line; a bead probe (*R* = 10,000 nm), black dashed line. The best fit models within a force range 0–100 nN are shown: the Hertz model (*R* = 20 nm), green line; the Sneddon model (half cone opening angle = 19 degrees), red curve; the Hertz model (*R* = 10,000 nm), purple line.

An example of the force-separation curve of tomato cell is shown in Figure 4a. Young's modulus *E* was calculated from the indentation part of the approach curve, *i.e.*, when force was greater than 0 nN, using two models [24]: the Hertz model (Equation (3), green and purple lines in Figure 4B) for both AFM probes, and the Sneddon model (Equation (4), red line in Figure 4B) for the tip of curvature radius *R* = 20 nm. The Sneddon model was not used for the bead probe due to clearly defined spherical tip geometry whereas in the case of the sharp conical tip both radius of curvature *R* and the half cone opening angle α needed for models were available:

(3) $$F=E\frac{4\sqrt{R}}{3(1-v^{2})}\delta^{\frac{3}{2}}$$

(4) $$F=E\frac{2\tan\alpha }{\pi (1-v^{2})}\delta^{2}$$

where R is the tip radius of curvature (nm), α is the half cone tip opening angle, δ is indentation (nm) and *v* is Poisson's ratio. In this experiment the nominal radiuses R provided by probe suppliers were taken. Taking into account a simplified model of plant cell as elastic cell wall filled with incompressible fluid Poisson's value *v* = 0.5 was assumed, which corresponds to an uncompressible material. The option of the best fit, within incrementally increasing range from zero force up to 100 nN with step of 10 nN, was chosen.

Stiffness was calculated in Matlab as the slope of the line drawn between 50 nN and 100 nN (green line in Figure 4).

### 2.4. Statistical Analysis

The experiment was performed on 19 cells; nine cells were measured with the sharp probe, 10 cells with the bead probe. No replicates were performed for cells. 250 force curves was collected for each cell in the way presented above, i.e., in the matrix of 25 points (10 force curves for each point) for the sharp probe and in a single point (250 force curves) for the bead probe. Elastic properties were determined separately for each curve and then mean value and standard deviation were then calculated.

## 3. Results and Discussion

**Figure 5.**
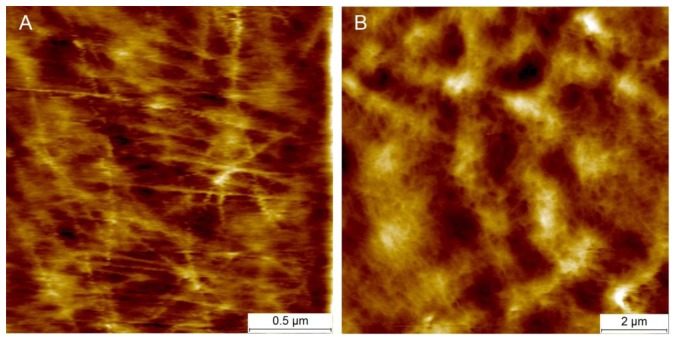
Examples of topography images of a living cell from tomato mesocarp obtained with AFM. (**A**) scan size 2 × 2 μm, Z height range 56 nm, (**B**) scan size 10 × 10 μm, Z height range 490 nm.

Figure 5 presents examples of AFM scans (topographic view) of the cell wall surface obtained directly from living tomato cells extracted from the mesocarp. This study showed for the first time, to the best of our knowledge, the possibility of nano-structure characterization of cell walls of individual living cells extracted from naturally grown fruit. The 2 × 2 μm scan (Figure 5A) shows cellulose microfibrils on the surface (Z height range 56 nm). In the 10 × 10 μm scan (Figure 5B), microfibrillar structure is hardly visible, and instead surface unevenness has been revealed (Z height range 490 nm). As is shown in Figures 3 and 5, it is possible with some effort to combine mechanical properties with topography imaging. This property has been already used by Peaucelle *et al.* [27] and Milani *et al.* [26] to study plant morphogenesis, however not for suspensions of extracted cells but for cells in tissue. For isolated cells, to make measurements effective the obstacle of firm fixation to a support should be overcome. Due to possible unevenness of the cell surface, cells could be detached from the support by the AFM probe, particularly when a larger area is scanned. Therefore, in further study to ensure more measurements effective it was decided to screen the areas of interest in the retract position of the AFM probe, which limited mechanical lateral affection.

To the best of our knowledge AFM nano-indentations have not been attempted previously on fruit cells suspended directly from parenchyma. He protocol that has been developed in this study for AFM nano-indentation of fruit cells is based on several known protocols: suspension-cultured cells of grapevine [25] and *Arabidopsis thaliana* [31], cells of living roots [29], shoot apical meristems cells [26,27] and a general protocol proposed by Radmacher [20,21].

**Figure 6.**
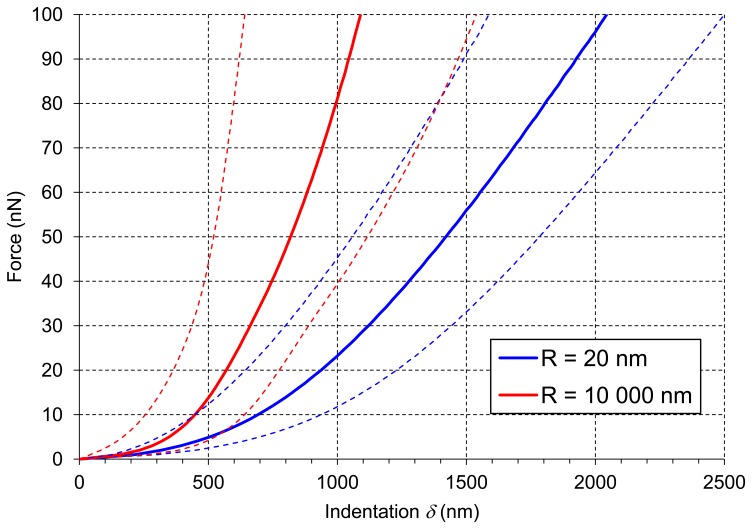
Averaged force-indentation curves (solid lines) recorded in the experiment for two AFM probes of different radius of curvature R. Dashed lines presents indentation ± standard deviations.

Figure 6 presents averaged force-indentation curves (N∼2,500) for the two AFM probes used in this experiment. For the sharper tip (*R* = 20 nm) indentation was about 2 μm, whereas for the bead tip (*R* = 10,000 nm) the averaged maximum indentation was only 1 μm (both at the force of 100 nN). Routier-Kierzkowska and Smith [18] stated that when the indenter diameter is similar to, or smaller than cell wall thickness, the measurement is influenced by both local cell wall compression and a more global deformation.

**Figure 7.**
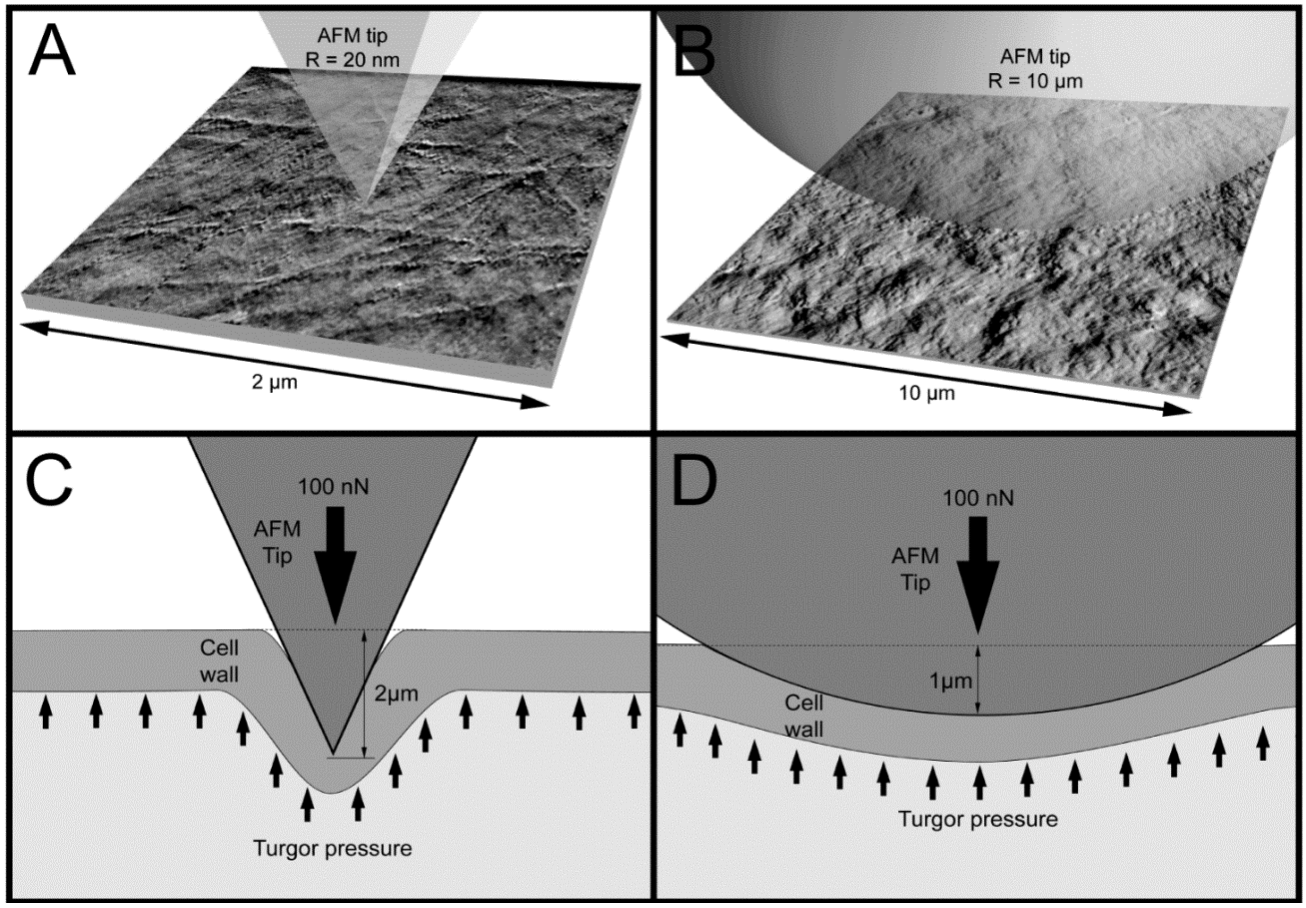
Schematic comparison of AFM tip radius, (**A**) *R* = 20 nm, (**B**) *R* = 10,000 nm, with cell wall structure of an intact tomato cell presented in adequate scales. Both pictures of cell walls in the background were obtained with AFM tip *R* = 20 nm. In (**A**) cellulose microfibrils of several nanometres in diameter are visible. In (**B**) cell wall microstructure is depicted. (**C**) and (**D**) show a proposed scheme of an indentation of fruit cells with two AFM probes *R* = 20 nm and *R* = 10,000 nm, respectively.

When the indenter diameter is much larger than the cell wall thickness, local compression of the cell wall can be neglected and the cell wall is rather bent and tensed (Figure 7D). A sharper tip also bends cell walls but may also cause penetration and local compression of the cell wall, which in summary leads to larger indentations to reach the desired force (Figure 7C). Penetration is possible because cellulose microfibrils are about 20–50 nm in diameter [11] and they are embedded in the pectic and hemicellulosic matrix (Figures 5A and 7A). This makes penetration among the cellulose microfibrils by the sharp probe possible. However, despite indentations that are deeper than the cell wall thickness, there were no visible disruptions of either cell wall or plasmalemma. Taking into account that the cell wall thickness is about 1 μm, indentation of 2 μm without visible disruption must cause cell wall local bending. In the case of the bead probe (*R* = 10,000 m) penetration is not expected because the dimension of the probe is much larger than packing of cellulose microfibrils (Figures 5B and 7B). Thus, probing cells with the bead probe causes some flattening and bending of the cell wall in the place of indentation. Presumably turgor affects the measurements in both cases due to bending of cell walls. However, particularly in the case of the bead probe, this could be significant due to a lack of penetration through the polysaccharide matrix and a significantly larger contact area. AFM force measurements also allow the application of smaller indentations (<100 nm), which as it was assumed by Radotić *et al.* [31] should avoid the turgor effect and allow the investigation of the mechanical properties of cell walls independently. However, since the texture of fruits depends on both cell wall elasticity and turgor this experiment was aimed at producing relatively large indentations. Results of the indentation to about 2 μm are presumably not affected by the underlying glass support, since the cells had dimensions >200 μm. Therefore, the indentation applied was about 1% of the cell height and meets the rule for the Hertz model that indentation should be no larger than 5–10% of the sample height.

**Figure 8.**
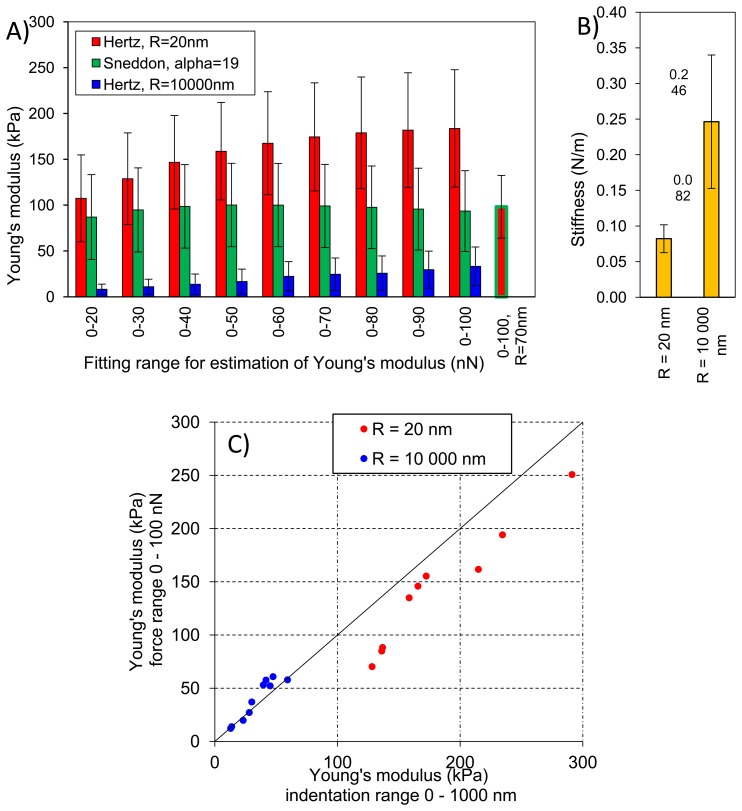
(**A**) Averaged Young's modulus calculated with the Hertz and the Sneddon models as a function of a force range used for model fitting. The last bar in (**A**) presents the Young's modulus form the Hertz model with corrected radius of curvature for sharper tip (*R* = 70 nm). (**B**) stiffness obtained by two probes of different radius of curvature R. (**C**) comparison of Young's modulus E calculated with the Hertz model within two regimes: indentation 0–1,000 nm and force 0–100 nN. Error bars on (**A**) and (**B**) are ± standard deviations.

Figure 8A presents averaged elastic properties of tomato cells extracted from mesocarp with respect to the models (Equations (3) and (4)) used for Young's modulus evaluation and the fitting range. Figure 8B presents also an average stiffness of cells obtained by these probes. For both probes used, Young's modulus from the Hertz model increased, which is noticeable despite the high SD, with increasing fitting range from about 100 kPa to 170 kPa and from 8 kPa to 33 kPa for the sharp probe and the bead probe, respectively. However, for the sharper probe (*R* = 20 nm) a tendency to plateau at higher loadings was also noted. For this probe, the Sneddon model has been applied too, showing lower estimated Young's modulus which was about 96 kPa and unambiguous independency on the fitting range.

This could be explained by better fitting of the Sneddon model to experimental data (as it is shown in Figure 4 as an example), particularly close to the contact point whereas the Hertz model, when the best fitting method has been applied, described more correctly the middle part of the fitting range. Although, the apparent fitting performance is similar for both models, the resulting Young's modulus is significantly different; at the 0–100 nN fitting range the Hertz model provided a double value in comparison to the Sneddon model. For the Hertz model the nominal radius of curvature of sharper probe was used, however while the conical probe was pushed into material its effective radius of curvature increased. Therefore, by a tip check procedure on the RS-12M titanium roughness sample proposed by Bruker, an effective tip radius of curvature at the distance of 250 nm from the tip end was estimated as about *R* = 70 nm. It was the maximal possible value obtained in this procedure: increasing distance from the tip end did not increase the estimated effective tip radius. The value *R* = 70 nm was then used to correct estimated Young's modulus from the Hertz model which gave 98 kPa at fitting range 0–100 nN (the last bar in Figure 8A). Taking into account that at the lowest fitting range (0–20 nN) Young's moduli from both models were very similar (about 100 kPa), after correction of the tip radius, the Hertz model provided a similar Young's modulus to the Sneddon model. One can conclude that using the sharp tip (ScanAsyst Fluid, nominal *R* = 20 nm), the mean Young' modulus of tomato cells (*N* = 9) was roughly about 100 kPa.

Figure 8C presents comparison of the Young's modulus calculated within the force range of 0–100 nN and within an indentation range of 0–1,000 nm. The relation is linear with slightly lower values of *E* when the loading range is used for model fitting, particularly in the case of the tip radius *R* = 20 nm. Figure 8C shows also that at the same indentation of 1,000 nm the two probes provide significantly different Young's moduli of cells.

In the case of the bead probe, due to its well defined geometry, the only the Hertz model was applied. Estimated Young's modulus was more than three times lower than for the sharp probe (Figure 8A). This must be result of the previously described different indentation through cell wall of the cell. The effect of indentation, i.e., penetration through material, is also visible for measured stiffness of cells (Figure 8B) The sharp tip measured cells as much less stiff (0.082 N/m) than the bead probe (0.246 N/m).

**Figure 9.**
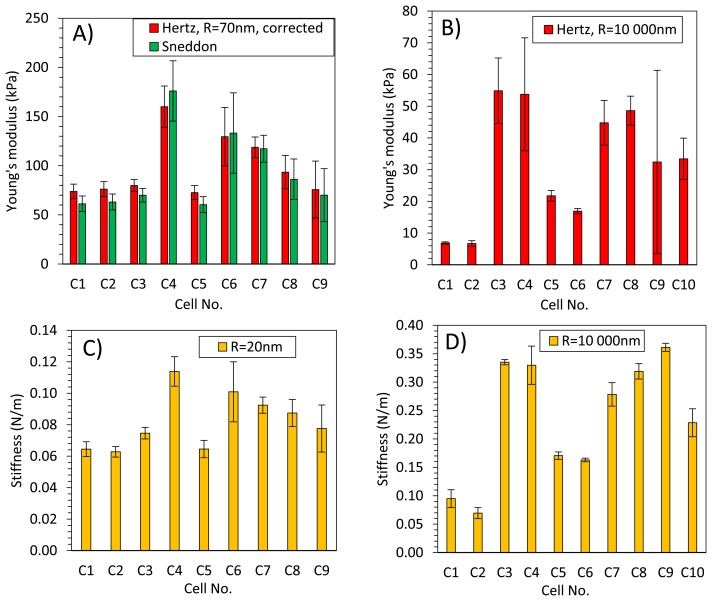
Elastic properties presented for individual cells. Error bars are ± one standard deviations. (**A**) Young's modulus obtained from the Hertz and Sneddon models for corrected tip radius *R* = 70 nm and the half cone opening angle = 19°, respectively; (**B**) Young's modulus obtained from the Hertz model for nominal tip radius *R* = 10,000 nm; (**C**) and (**D**) Stiffness obtained in the experiment with tip radius of curvature *R* = 20 nm and *R* = 10,000 nm, respectively. In the case of (**A**) and (**B**) force fitting range was 0–100 nN.

Figure 9 presents elastic properties for individual cells with respective standard deviations (SD) to show variability of properties within a very small volume of tomato pericarp (please refer to the Experimental section for details). These results were compared with geometrical size of cells however no clear relationship was found (data not shown), which suggests that cells are large enough to validate the assumption that the sample has an infinite size comparing to the tip geometry. Figure 8 shows that total standard deviation of Young's modulus form the Hertz model was about 35% and 73% of the mean value for the sharp and the bead probe, respectively. Comparing this with Figure 9 it is clearly seen that for individual cells, standard deviation was much lower; roughly about 15% and 21% for the two probes, respectively. However differences among cells regarding their elastic properties was >100%. The same summary could be made for the Sneddon model and stiffness. From these observations, and previous results by Wang *et al.* [32], one can conclude that there would be significant difference regarding elastic properties among individual cells from the same fruit, moreover from a relatively small volume of the same tissue.

The results described above show that the apparent Young's modulus of cells of fully mature tomato mesocarp could be of kilopascals magnitude (depending on the AFM probe used). Unfortunately, there are no studies available to compare these values since most experiments have been oriented on cell wall properties only. Radotić *et al.* [31] who studied suspension-cultured cells of *Arabidopsis thaliana* showed that cell wall Young's modulus (calculated from the Hertz model in a very similar way as in our study) varied between 0.1–1 MPa and after 20 days of culture was less than 200 kPa. In that study it was assumed that indentation <100 nm provides information on cell wall properties only. Taking into account that they used an AFM probe with similar radius of curvature (20 nm), the result for *Arabidopsis thaliana* cells was of the same order as for cells from tomato fruits obtained in our experiment. However it must be emphasized that in the case of our experiment, indentation was much larger and due to fact that the Hertz and Sneddon models require an assumption of sample homogeneity, the Young's modulus does not reflect the cell wall properties alone and convolutes intracellular pressure too. Significantly different results to Radotić *et al.* [31] were found earlier by Blewett *et al.* [17], who estimated the cell wall Young's modulus in the range of 100–2,300 MPa. The maximum value (2.3 GPa) was then accepted by Wang *et al.* [33] and next by Dintwa *et al.* [12] to model the compression of single tomato suspension cells from a root radicle callus. Wang *et al.* [33] found that the elastic moduli cell wall of pericarp cells from commercially grown tomato fruits (the same type as in our experiments) was in the range of 30–80 MPa. For other fruits; Wu and Pitts [34] assumed the Young's modulus for apple cell walls to be 26.4 MPa, whereas Davis *et al.* [35] estimated that potato cell wall modulus to be 105 MPa.

The studies presented above were aimed on estimation of cell wall elastic properties whereas taking into account thickness of cell wall of tomato mesocarp, which is presumably about 1 μm, and a weakness of the wall for bending, indentation 1–2 μm performed with AFM causes an additional effect from turgor (please refer to the schematic view of cell wall deformation in Figure 7C,D). Therefore when the elastic properties of cells are estimated directly from AFM force curves, particularly when the Hertz or Sneddon models are used, the Young's modulus is a convolution of cell wall elasticity and the value of turgor inside the cell. Eventually, cell wall Young's modulus could be deconvoluted with help of a shell-like model [19,36] or by applying very small indentations [31].

## 4. Conclusions

This study shows that atomic force microscopy could be used for evaluation of the elastic properties of cells extracted from naturally grown fruit pericarp. The indentation of cells could be up to 2 μm, which probably senses both cell wall and turgor properties. Based on a literature review, to study only the cell walls' properties one could apply smaller indentations than the cell wall thickness or apply a shell-like model, however further study is required to state this unambiguously.

There is significant influence of the AFM tip radius of curvature on elastic properties calculated from the Hertz model; a larger tip radius of curvature provides a lower apparent Young's modulus of cells. When the AFM probe has a radius of curvature similar to the dimensions of the cell wall polysaccharides, indentation must be larger to achieve a certain force. The bead type probe, of a diameter much larger than the nano-structural features of the cell walls, bends the cell wall without penetration. This result shows that the nano-mechanical properties of plant cells sensed with a probe of nanometer radius of curvature could significantly differ from the micro-mechanical properties sensed with a probe of micrometer radius of curvature. It was observed also that correction of tip radius of curvature due to penetration of the tip in material allows for closer estimation of elasticity properties from the Hertz and Sneddon models.

The experiments showed a great cell variability regarding the elastic modulus (>100%), despite the fact we were sampling a relatively small volume of the tomato mesocarp. This means that when dealing with such a large microstructural variability to explain the macroproperties of fruits, developing a robust technique for the investigation of cell and cell wall properties is of great importance. AFM is now a popular and commercially available device which, considering the results of this study, offers new opportunities for effective characterization of both structure and mechanics of fruits on the cell (micro) and cell wall (nano) level. By combining AFM topography and nano-indentation of living fruit cells it will be possible to study cells' mechanical properties, concerning processes like postharvest cell wall degradation and turgor change.
